# Novel biostimulants-mediate tolerance to drought stress in *Phaseolus vulgaris* plants by optimizing osmoprotectants and antioxidant defense systems

**DOI:** 10.1186/s40529-025-00483-x

**Published:** 2025-10-20

**Authors:** Hussein E. E. Belal, Amr Elkelish, Mohamed M. Zaid, Abdulrahman Alhudhaibi, Mohamed S. A. El-Roby, Yasmine H. Abd Elmohsen, Amany H. A. Abeed, Celestin Ukozehasi, Mostafa M. Rady, Ali A. S. Sayed

**Affiliations:** 1 Botany Department, Faculty of Agriculture, Fayoum University, Fayoum, 63514 Egypt; 2 Department of Biology, College of Science, Imam Mohammad Ibn Saud Islamic University (IMSIU), Riyadh, 11623 Saudi Arabia; 3 Vegetable Research Department, National Research Centre, P.O. Box 12622, Dokki, Giza Egypt; 4 Department of Botany and Microbiology, Faculty of Science, Assiut University, Assiut, 71516 Egypt; 5 Department of Crop Science, School of Agriculture and Food Sciences, University of Rwanda, Kigali, 6605 Rwanda

**Keywords:** Drought stress, *Phaseolus vulgaris*, Biostimulants, Lemon fruit juice, Bee honey, Antioxidant defense, Crop productivity, Irrigation regimes

## Abstract

**Background:**

Drought stress is a major constraint to the growth and productivity of *Phaseolus vulgaris*, particularly in dry regions. Utilizing natural biostimulants offers an eco-friendly approach to enhancing drought tolerance by modulating plant physio-biochemical responses. This study investigates the effectiveness of diluted lemon fruit juice (DLFJ) and diluted bee honey (DBH) as foliar biostimulants to mitigate drought-induced stress in *P. vulgaris* under two irrigation regimes: full (100% ETc) and deficit irrigation (60% ETc).

**Results:**

Both DLFJ and DBH treatments significantly enhanced photosynthetic efficiency, relative water content (RWC), membrane stability index (MSI), osmoprotectant levels, antioxidant activity, and nutrient accumulation under drought conditions. Among all treatments (4% and 8% DBH, and 3% and 6% DLFJ), DBH-4% was the most effective. It significantly increased chlorophyll and carotenoid contents, photosynthetic efficiency, leaf integrity (RWC and MSI), osmoprotectant levels, antioxidant activities, and green pod yield under 60% ETc compared to untreated controls. It also markedly reduced oxidative stress markers (e.g., malondialdehyde and hydrogen peroxide) and further boosted enzyme activities, including superoxide dismutase, catalase, ascorbate peroxidase, and glutathione reductase.

**Conclusions:**

These findings demonstrate that DBH-4% is a promising and sustainable biostimulant for improving drought resilience in *P. vulgaris*. Its rich content of antioxidants and osmoprotectants confers significant physiological and agronomic gains under water-limited conditions.

## Background

Common beans, *Phaseolus vulgaris* L., are historical legume crops that hold considerable interest in various developing countries, including Egypt, for local consumption and as an export commodity (Thabet et al. 2024; Elkelish and Abu-Elsaoud 2024a). It is a rich source of protein, dietary fiber, starch, and vitamins (Rady et al. 2023a). As the world’s need for safe and high-quality food crops increases, this challenge must be met by exploring efficient and eco-friendly agronomic systems that can be easily and successfully implemented worldwide (Pascual et al. 2018; Mishra et al. 2020). Moreover, agricultural production strives to enhance crop quality through technological advancements that foster production sustainability by substantially decreasing the application of agrochemicals, such as fertilizers and pesticides. Consequently, biostimulants have gained popularity as plant growth-promoting preparations that positively impact plant growth, development, and yields (Rady et al. 2021, 2023a, b; Schierenbeck et al. 2023).

Currently, high-efficiency NBS application has been proven to enhance physio-biochemical processes in plants by upregulating phytohormones. This reflects reinforced root systems to optimize the uptake, transport, and use of nutrients, all of which contribute to enhancing crop yields and qualities (Thabet et al. 2021; Makhadmeh et al. 2022; Rady et al. 2023a, b). They improve the plant’s resistance to various abiotic stressors by modifying and promoting the synthesis and activity of antioxidant enzymes to reinforce the growth, nutrient uptake, and crop yields due to promoting plant stress tolerance (Semida et al. 2019; Rady et al. 2021, 2023a, b; Ávila-Pozo et al. 2023). NBS are recognized for their activity as biostimulants, which are considered eco-friendly and can maximize crop productivity and sustainability (Bhardwaj et al. 2022). Certain studies have been performed using propolis and maize grain embryo extracts (Semida and Rady 2014; Desoky et al. 2021; Hemida et al. 2024), *Moringa oleifera* leaf extracts (Rady and Mohamed 2015; Abd El-Mageed et al. 2017), licorice root extracts (Rady et al. 2019), garlic and onion extracts (Rady et al. 2023b), natural bee-honey-based NBS (Semida et al. 2019), and lemon fruit juice (Rady et al. 2023a) to promote a plant’s ability to tolerate stress by optimizing osmoprotectant (OSPs) and antioxidant defense systems. Therefore, NBS could be promising for areas with degraded agriculture and unpredictable weather patterns (Abd El-Mageed et al. 2017; Rady et al. 2021, 2023a, b).

DLFJ is a valuable source of organic acids (e.g., citric, with the highest percentage of ascorbic and malic acids), various (micro- and macro-) mineral nutrients, vitamins, sugars, and carbohydrates (Abd El et al. 2024; Elkelish and Abu-Elsaoud 2024b). In addition, DLFJ, as one of the NBS, contains essential bioactive compounds (EBCs), including phenolic acids, flavones, and flavonoids, and has high antioxidant activity (Klimek-Szczykutowicz et al. 2020). Under 1.0 mM Cd stress, application of 5% DLFJ has been shown to optimize *P. vulgaris* seed germination, photosynthetic pigments and efficiency, RWC, MSI, OSPs, and various antioxidant activities (both non-enzymatic and enzymatic). These positive findings positively impacted growth, seedling biomass, productivity, and yield quality. Conversely, DLFJ minimized oxidative stress indices (e.g., O_2_^•−^ and H_2_O_2_), stress damage indices (e.g., MDA and EL), Cd translocation factor, and plant Cd content (Rady et al. 2023a).

BH, an aqueous dispersion, contains huge inorganic and organic molecules, including organic acids, polysaccharides, soluble sugars, flavonoids, mineral nutrients, proteins, vitamins, fatty acids, amino acids, and enzymes (Saxena et al. 2010; Isla et al. 2011; Semida et al. 2019; Rady et al. 2021, 2023b; Tarfayah et al. 2023). It contains the enzyme catalase with ROS-eliminating properties, glucose oxidase that converts glucose to H_2_O_2,_ and gluconic acid, which gives it antimicrobial properties (Chirsanova et al. 2021). DBH remarkably eliminates DPPH-scavenging radicals. It elicits physio-biochemical and antioxidant modifications to minimize ROS levels and optimize stress tolerance in crop plants (Semida et al. 2019; Rady et al. 2021, 2023b; Tarfayah et al. 2023). In addition, the flavonoids and enzymes containing DBH impede the peroxidation process and contribute to ROS removal (Saxena et al. 2010). Under different stresses, DBH application minimizes oxidative stress indices (e.g., O_2_^•−^ and H_2_O_2_) and stress damage indices (e.g., MDA and EL), reflecting in enhanced plant growth, productivity, yield quality, regulation of photosynthesis, hormonal and nutritional balances, leaf integrity, OSPs levels, different antioxidant activities, and enzymatic gene expression (Semida et al. 2019; Abou-Sreea et al. 2021; Rady et al. 2021, 2023b; Tarfayah et al. 2023).

Using these NBS is crucial because their EBCs can penetrate plant leaf cells when applied as a foliar spray, enhancing essential mechanisms for plant growth, productivity, and high-quality yields. So far, very few studies have been conducted on applying DLFJ and DBH, as NBS, to optimize plant growth and yield quality under stress. Based on previous works (Semida et al. 2019; Abou-Sreea et al. 2021; Alghamdi et al. 2022; Rady et al. 2023a), the hypothesis tested is that foliar spray of DLFJ and DBH for *P. vulgaris* plants grown under two irrigation regimes (100 and 60% ETc) will enhance the photosynthesis, nutrient uptake, OSPs levels, different antioxidants, growth and yield quality. Several studies have examined the effects of biostimulants on drought stress mitigation in legumes. For instance, Moringa oleifera leaf extract has been reported to improve drought resilience in Phaseolus vulgaris by enhancing antioxidant enzyme activity and osmotic adjustment (Rady et al., 2015). Similarly, biostimulants derived from seaweed extracts have been found to enhance growth and yield in legumes under water stress conditions (Pascual et al. 2018). While these studies highlight the potential of biostimulants, they primarily focus on plant-based extracts or synthetic compounds, with limited exploration of food-based natural biostimulants like diluted lemon fruit juice (DLFJ) and diluted bee honey (DBH). Unlike synthetic or processed plant extracts, DBH and DLFJ provide a unique composition of natural antioxidants, osmoprotectants, and essential bioactive compounds, which may confer a broader spectrum of stress resilience mechanisms. However, to our knowledge, no previous study has systematically evaluated the efficacy of these natural biostimulants in Phaseolus vulgaris under controlled drought stress conditions. This study fills this critical gap by assessing their impact on photosynthetic performance, osmoprotectant accumulation, antioxidant defenses, and overall plant productivity in a field setting. Therefore, this work aimed to explore the impacts of foliar spraying with DLFJ (3 and 6%) or DBH (4 and 8%) on the following estimations in drought-stressed *P. vulgaris* plants: (I) photosynthetic parameters and different (macro- and micro-) mineral nutrients, (II) leaf integrity, ROS (e.g., O_2_^•−^ and H_2_O_2_) and stress damage indices (e.g., MDA and EL), (III) various OSPs (osmotic adaptability) and different antioxidant (non-enzymatic and enzymatic) activities, and (IV) *P. vulgaris* plant growth, productivity, and pod yield quality.

## Materials and methods

### Experimental location and soil analysis

A plot of clayey soil with an area of about 700 m^2^ was allocated on a private farm (Al-Wasta District, Beni Suef; 29°19’48"N, 31°11’13"E, Egypt) to carry out a field study for the 1st early summer season (2023). The 2nd season (2024) was conducted on another soil plot with the same area as the first season’s plot.

Table 1 displays the average of two-season climatic conditions at the study site. Soil samples were analyzed to evaluate the physicochemical properties (Page et al. 1982; Klute 1986). Table 2 shows soil analysis data, including soil ECe = 1.58 dS m^−1^ (non-saline soil) (Page et al. 1982).

**Table 1:** Average weather data of Al-Wasta District (Beni Suef) during the two growing seasons

Month	Day °C	Night °C	ARH (%)	AWS (km h^–1^)	AP (mm d^–1^)
2022 season					
October	30.5	16.3	59.4	17.2	0.45
November	28.0	14.0	47.9	17.8	0.50
December	24.5	12.5	44.8	18.5	0.60
2023 season					
October	30.0	16.0	59.6	17.5	0.54
November	27.5	13.6	46.2	17.9	0.62
December	23.4	12.0	44.5	19.0	0.66

Day °C; average day temperature, Night °C; average night temperature, ARH; average relative humidity, AWS; average wind speed, and AP; average precipitation

**Table 2:** Physicochemical properties of the before experimentation

Soil Texture	pH (in soil paste at 25 °C)	ECe (in soil paste extract at 25 °C)	CaCO_3_	Na^+^ conc.	Ca + Mg conc.	SAR	OM	FC	AW
		dS m^−1^	%	Meq L^−1^			%		
Clay	7.94	1.58	3.69	3.30	12.00	1.34	0.96	27.8	16.4

SAR; sodium adsorption ratio, OM; organic matter, FC; field capacity, AW; available water, and conc.; concentration

### Seed sowing, treatments, and trial layout

*Phaseolus vulgaris* seeds (cultivar Sabaris, Bayer Seminis) were provided by the Egyptian Green Force Company for this study. They were disinfected with NaClO solution (1.0%) for 120 s. They were then surface-cleaned from the disinfection solution with deionized water (D-H_2_O) and air-dried overnight. For both summer seasons (2023 and 2024), the seeds were sown on 25 February in the hills. The distances were 20 cm between hills in each row and 70 cm between two rows, and each plot (experimental unit) was 10.5 m^2^ (length 3.50 m × width 3.00 m).

Both seasonal experiments were arranged in a split-plot design with two main factors (watering regimes × NBS). The main factor was the watering regimes (100% and 60% ETc). Under each watering regime, 5 NBS treatments were applied in a completely randomized design, replicating each treatment in 6 randomized plots. The NBS treatments were (1) control (spraying plants with D-H_2_O), (2) foliar spraying with DLFJ at 3% (8.82% TSS) of juice, (3) foliar spraying with DLFJ at 6% TSS (8.82%) of juice, (4) foliar spraying with DBH at 4% (83.8% TSS) of honey, and (5) foliar spraying with DBH at 8% TSS (83.8%) of honey. The DLFJ and DBH concentrations used in this study were selected based on a preliminary study (data not shown).

The soil was fertilized with 300 kg ammonium nitrate (33% N) + 200 kg CaH_6_O_9_P_2_ (15% P_2_O_5_) + 200 kg K_2_SO_4_ (50% K_2_O) ha^−1^. Half of the N, P, and K doses were applied during soil preparation for planting, and the other halves were added immediately before the 2nd irrigation. A distance of 2 m was left between the two main soil strips of the two watering regimes to prevent interference between the IR treatments. When the plants reached 3, 4, and 5 weeks of age, their leaves were sprayed with NBS solutions. The DLFJ solutions were prepared by adding 3 or 6 mL of juice L^−1^ of D-H_2_O and mixing well. Also, the DBH solutions were prepared by adding 4 or 8 mL of fresh clover bee honey L^−1^ of D-H_2_O and mixing well. In the early morning, the sprays were performed immediately after preparing the DLFJ and DBH solutions. The spray solutions were thoroughly mixed with surfactant (Tween-20 at 0.1%) for optimal penetration into leaf tissues. Within each IR strip, a distance of 1 m was left among the plots to prevent interference with the NBS treatments. Lemon fruit juice and raw clover bee honey samples were analyzed for their physicochemical properties (A.O.A.C. 2000), and the data are presented in Table 3.

**Table 3:** Chemical characteristics of the lemon fruit juice and Raw clover bee honey

Components	Units	Lemon fruit juice	Bee honey
pH		3.10 ± 0.20	4.38 ± 0.24
Organic acids	%	5.92 ± 0.31	0.51 ± 0.03
Total soluble solids	°Brix	8.82 ± 0.42	83.8 ± 4.24
Total free amino acids	%	4.13 ± 0.22	0.35 ± 0.02
Total soluble sugars		1.18 ± 0.05	81.3 ± 4.02
Fructose		0.54 ± 0.03	32.8 ± 2.31
Glucose		0.49 ± 0.02	39.4 ± 2.77
Sucrose		0.03 ± 0.00	2.48 ± 0.20
Maltose		traces	4.55 ± 0.26
Vitamin C		0.03 ± 0.00	0.05 ± 0.00
Total anthocyanins		0.01 ± 0.00	0.05 ± 0.00
Total phenols		0.12 ± 0.01	0.13 ± 0.00
Ash		0.34 ± 0.01	0.39 ± 0.02
Calcium	mg g^−1^ FW	0.08 ± 0.00	0.09 ± 0.00
Magnesium		0.07 ± 0.00	0.09 ± 0.00
Phosphorus		0.07 ± 0.00	0.08 ± 0.00
Potassium		0.07 ± 0.03	0.08 ± 0.00
Iodine		0.02 ± 0.00	0.04 ± 0.00
Antioxidant activity	mM Trolox eq. L^−1^	17.2 ± 0.78	19.8 ± 0.92

eq.; equivalent

The IR treatments (IR-1;100% ETc and IR-2; 60% ETc) were calculated by the equation ETc = ETo × Kc, where ETc and ETo are Crop and Reference evapotranspiration (mm day^−1^), respectively. ETo was computed from weather data (daily) by the equation Penman-Monteith and Kc = Crop coefficient, which varies across the growth stages of green beans (Allen et al. 1998). Irrigation volume of 6600 or 3960 m^3^ was achieved ha^−1^ for IR-1 or IR-2, respectively. Every 10 days, 940 or 565 m^3^ was achieved ha^−1^ for the IR-1 or IR-2 treatments, respectively.

Table 4 presents the chemical composition of the irrigation water used.

**Table 4:** Chemical **composition** of irrigation water

Concentration of ions (meq L^−1^)								EC (dS m^−1^)	pH	SAR
CO_3_^2−^	HCO_3_^−^	SO_4_^2−^	Cl^−^	Mg^2+^	Ca^2+^	K^+^	Na^+^			
0.00	2.11	3.16	10.2	1.72	5.30	1.52	3.34	1.48	7.36	2.44

EC; electrical conductivity, and SAR; Sodium adsorption ratio

Considering the full recommendation of the Egyptian ARC, weeds were controlled manually and by hoeing twice a week. Pathogens were controlled biologically, with each kg of seeds mixed well with 2.5 g each of Bio-Arc + Bio-Zeid (6.0 + 2.5% *Bacillus megaterium* + *Trichoderma album*; 2.5 × 107 cells g^−1^ + 10 × 106 spores g^−1^, respectively).

### Preparation of biostimulants

Lemon fruits (a local variety) were collected from an orchard beside the experimental farm. Once the lemon fruits arrived at the laboratory, they were squeezed to extract the juice, which was then filtered twice: first through a muslin cloth and then through Whatman No. 1 filter paper. The value of TSS (8.82 °Brix) in lemon juice was measured and recorded using a refractometer. Once the lemon juice was filtered, it was diluted by adding 3.0 or 6.0 mL of juice L^−1^ D-H_2_O and used immediately. These concentrations (3.0 and 6.0 mL L^−1^) were the best used in a preliminary trial (data not shown). Egyptian clover bee honey was secured from the apiaries of the Egyptian Ministry of Agriculture. The value of TSS (83.8%) in the bee honey was measured and recorded by a refractometer. The bee honey was diluted at 4.0 and 8.0 mL L^−1^ D-H_2_O and used immediately. These concentrations (4.0 and 8.0 mL L^−1^) were the best used in a preliminary study (data not shown).

### Estimation of photosynthesis parameters

The total leaf TChls and TCars content (mg g^−1^ fresh tissue) were measured using the method in (Wellburn 1994), with pigments extracted in 80% acetone. Absorbance was recorded at 470, 653, and 666 nm. The relative chlorophyll content of the two uppermost leaves was assessed using a SPAD-502 Chl-meter (Minolta, Japan), while photosynthetic efficiency (Fv/Fm) was evaluated as in (Maxwell and Johnson 2000) using a fluorometer (PAM-2000, Heinz-Walz). Chlorophyll fluorescence was measured under red actinic light (627 nm) at 3500 µM PAR m^− 2^ s^−1^ for 1 s. Fluorescence parameters Fv/F0, Fv/Fm, and PIABS were recorded from four fully grown leaves per replicate (Clark et al. 2000).

### Estimation of nutrient contents

Nutrient (including Se) contents were determined in dried *P. vulgaris* leaf powder. The micro-Kjeldahl system evaluated the total N content (A.O.A.C. 2000). Colorimetrically, after extraction by NaHCO_3_, the stannous chloride-ammonium molybdate reagent was applied to assess P content (Olsen et al. 1954; King and Wotton 1957). An ELE-Flame Photometer (Leighton Buzzard, UK) system was applied to evaluate K^+^ and Na^+^ contents. An Atomic Absorption Spectrophotometry system was also applied to assess Ca^2+^, Mg, and micronutrient contents (Chapman 1965). The dried leaf samples (0.5 g) were wetted for Se by adding 5.0 mL of HNO_3_. The mixture was stirred slightly after carefully adding H_2_O_2_ (4 ml of 33%). The mixture was heated to produce a strong effervescence. After cooling the solution, it was filtered and washed with HCl (5 mL, density of 1.18 g mL^−1^). The solution was then reached 25 mL with D-H_2_O (Pequerul et al. 1993). Se was quantified as µg g^−1^ DW after obtaining the reads by ICP (Induction Plasma Spectrometer, Thermo Jarrell Ash brand), IRIS advantage model (A.O.A.C. 2000); the 984.27 methods.

### Determination of leaf tissue integrity and oxidant levels

The leaf RWC (%), MSI (%), and EL (%) were measured by applying the protocols of (Osman and Rady 2014), (Rady 2011), and (Rady and Rehman 2016), respectively. Leaf tissue RWC was evaluated using discs (2 cm diameter) prepared from a midrib-free leaf blade and weighted to record fresh mass. Then, the discs were saturated entirely in D-H_2_O in the dark for a whole day. Before recording the turgid mass, the discs’ surfaces were carefully freed from D-H_2_O. After that, they were dried for 2 days at 70 °C to obtain the dry mass value. Then, RWC was calculated:

$$\:RWC\:\left(\%\right)=\:\left[\frac{(fresh\:mass-dry\:mass)}{(turgid\:mass-dry\:mass)}\right]\times\:100$$

To estimate the MSI, 0.2 g midribs-free leaf blades were taken twice in two test tubes containing 10 cm^3^ D-H_2_O. A tube was heated at 40 °C for ½ h, and the solution’s electrical conductivity was taken (EC_1_). The 2nd tube was boiled at 100 °C for 10 min, and EC_2_ was taken. Then, the MSI was calculated:

$$\:MSI\:\left(\%\right)=\:\left[1-\left(\frac{{EC}_{1}}{{EC}_{2}}\right)\right]\times\:100$$

Midribs-free leaf blades were identified to prepare 20 discs to estimate the EL (%). The D-H_2_O was utilized to soak the discs, and EC_1_ was taken. After heating the contents (discs + D-H_2_O) at 45–55 °C for ½ h and then boiling them for 10 min, EC_2_ and EC_3_ were taken, respectively. Then, the EL was calculated:

$$\:EL\:\left(\%\right)=\:\left[\frac{({EC}_{2}-{EC}_{1})}{{EC}_{3}}\right]\times\:100$$

The levels of oxidative stress were assessed by measuring malondialdehyde (MDA) and hydrogen peroxide (H_2_O_2_) content in the leaves of *Phaseolus vulgaris* plants. Both markers were measured spectrophotometrically, with absorbance recorded at appropriate wavelengths according to (Tsikas 2017; Mihailova et al. 2023) methods, respectively.

$$\eqalign{&{\rm{MDA}}\left( {\mu {\rm{mol}}{{\rm{g}}^{ - 1}}{\rm{FW}}} \right) = 6.46\left( {{A_{532}} - 600} \right) - \cr& 0.56> \times >{A_{450}} \cr} $$

$$\eqalign{& {{\rm{H}}_2}{{\rm{O}}_2}\left( {\mu {\rm{mol}}{{\rm{g}}^{ - 1}}{\rm{FW}}} \right) = \cr& {A_{390}}/Standard>curve>slope> \cr} $$

The production rate of O_2_^•−^ was determined via spectrophotometry (Boveris 1984), requiring 2 g of plant tissue for these measurements.

### Estimation of antioxidant compounds

The detailed procedures of (Kampfenkel et al. 1995) and (Griffith 1980) were practiced to evaluate both contents (µM g^−1^ FW) of AsA and GSH, respectively. Nagy et al. (Nagy et al. 2017) methods were harnessed to determine α-tocopherol content (µg g^−1^ FW). After extraction, 20–200 µg n-hexane ml^−1^ as standard solutions were prepared, using R-α-tocopherol, to saponify samples at 80 °C for 40 min, and then direct cooling was performed. Three further extractions were performed using a mixture of n-hexane and ethyl acetate. After washing the combination with D-H_2_O and filtering the organic phase, the filtrate was evaporated to dryness. The residues were dissolved by mobile phase (n-hexane, HPLC grade) to estimate α.tocopherol content by HPLC system (Agilent 1100/1200 Series, USA), or stored at − 20 °C.

### Determination of antioxidant compounds and total antioxidant activity (TAA)

Methanol extracts were prepared from freeze-dried leaf samples by macerating 1.5 g of the sample in 15 mL of 100% methanol (Guedes et al. 2022). After three rounds of extraction, total phenolic content (mg gallic acid equivalents per gram dry weight) was measured following the method of Mongkolsilp et al. (Mongkolsilp et al. 2004). Total flavonoid content was assessed spectrophotometrically (Moein et al. 2008) with minor modifications. In brief, 4 µl of extract was mixed with a solution (10 µl of 1 M potassium acetate, 10 µl of 10% AlCl_3_, and 176 µl of H_2_O) and kept at 26 °C for 30 min. Mixture absorbance was then determined at 405 nm. The results were expressed as mg of quercetin equivalent per 1 gram of extract (mg QE g^−1^ fresh weight). Total antioxidant activity (%) in *P. vulgaris* was estimated by the DPPH-free radical scavenging activity test (Brand-Williams et al. 1995).

### Determination of osmoprotectant contents

Spectrophotometrically, the Irigoyen et al. (Irigoyen et al. 1992) procedure was practised to evaluate TS sugar content. After centrifugation, newly prepared anthrone was added to the supernatant, boiled for 10 min, and the OD was recorded at 625 nm. Spectrophotometrically, GB content was evaluated (Subbarao et al. 1999). A 0.5 g liquid N-frozen sample was crushed in a 4 mL mixture of 12 methanol: 5 chloroform: 3 water. Overnight, the homogenate was incubated at 4 °C. Using ion exchange resin (BioRad AG1-X8), the upper methanol phase was taken (1 mL), purified, and centrifuged (5000 ×g for 10 min). After filtration, the supernatant was loaded into the HPLC system (Agilent 1100/1200 Series, USA). The content of GB was then detected at 230 nm and quantified using GB standard curves. Bradford’s method (Bradford 1976) was harnessed to evaluate soluble protein content. Spectrophotometrically, proline content was assessed (Bates et al. 1973). After conducting an extract centrifugation, the supernatant was treated with a newly prepared acid-ninhydrin solution. After incubating the mixture at 90 °C for 30 min, the reaction was stopped on ice. A toluene solution was used for an additional extraction to obtain the toluene phase, which was read at 520 nm.

### Determination of enzymatic activity

Leaf samples (200 mg) were soaked in liquid N_2_ and homogenized in 2.0 mL of extraction buffer: 100 mM potassium phosphate (pH 7.8), 0.1 mM ethylenediaminetetraacetic acid (EDTA), and 10 mM ascorbic acid. The supernatant collected after homogenizing the sample and centrifuging (15,000 g for 10 min at 4 °C) of the homogenate was exploited. The activity of SOD, GR, CAT, and APX was evaluated by exploiting the procedures of (Giannopolitis and Ries 1977), (Smith et al. 1988), (Ali et al. 2019), and (Asada 2006), respectively. All enzyme activities are presented as “unit g^−1^ protein”.

### Determination of growth, green pod yield, and pod quality

Eight weeks after sowing (on 23 and 22 April 2023 and 2024, respectively), leaves were counted for each plant, and the area of plant leaves was taken (cm^2^) with a Digital-Planix 7 planimeter. After weighing the shoots to obtain fresh weights (g), they were exposed to 70 ± 2°C to dry until the weights were stable. Nine weeks after planting (on 30 and 29 April 2023 and 2024, respectively), green pods were harvested from *P. vulgaris* plants several times every three days, and the pods were counted and weighed for number and yield [g plant^−1^ and ton ha^−1^].

The micro-Kjeldahl system evaluated the total pod N% (A.O.A.C. 2000). Pod protein content (%) was evaluated using the equation N% × 6.25 = pod protein%. Pod fiber (%) was assessed by applying the procedures of Rai and Mudgal (Rai and Mudgal 1988). Pod vitamin C (AsA) content (mg g^−1^ FW) was estimated (Law et al. 1983). Pod NO_3_^−^ content (mg g^−1^ DW) was evaluated. Dried, ground samples were analyzed for NO_3_^–^ content, based on the extraction of the sample NO_3_^–^-N in water for 1 h at 45 °C (Cataldo et al. 1975). With the help of salicylic acid + sulfuric acid + NaOH solution added to the filtrate after centrifugation, the OD of NO_3_^–^-N was taken at 410 nm utilizing a spectrophotometer (Shimadzu UV-160 A). Potassium nitrate was utilized as a reference and 4.42 was applied as a coefficient.

### Statistical analysis

Error variance homogeneity for data from 2023 to 2024 was tested (Gomez and Gomez 1984) before the performance of the data statistical analysis using a two-way ANOVA. The GLM procedure of Gen STAT (version 11) (VSN International Ltd., Oxford, UK) was applied, and differences between means were tested using the LSD test (Waller and Duncan 1969) at *p* ≤ 0.05.

## Results

### Diluted lemon fruit juice (DLFJ) and bee honey (DBH) as novel biostimulants (NBS)

As shown in Table 3, The lemon fruit juice and bee honey were characterized by high levels of essential bioactive compounds (EBCs), including soluble sugars, free amino acids, vitamin C, phenolic compounds, and key minerals such as K, Ca, and Mg. Their antioxidant activity (17.2 and 19.8 mM Trolox eq., respectively) and low pH values (3.10 for lemon juice and 4.38 for bee honey) indicate potential for modulating stress responses. These findings justify their use as novel biostimulants (NBS), applied as foliar sprays at 3% and 6% diluted lemon fruit juice (DLFJ-3% and DLFJ-6%), and 4% and 8% diluted bee honey (DBH-4% and DBH-8%), to assess their effects on *P. vulgaris* under drought (60% ETc) and full watering (100% ETc) regimes.

### Effects of NBS on photosynthetic parameters of drought-stressed *P. vulgaris* plants

Drought stress (IR-2) significantly reduced photosynthetic traits in *P. vulgaris*, with total chlorophylls (TChls), carotenoids (TCars), SPAD values, Fv/Fm, and PIABS showing declines of 35.2–38.5% compared to full irrigation (IR-1) (Table 5). Application of either DLFJ or DBH improved all photosynthetic indices under both irrigation regimes. DBH treatments were more effective than DLFJ, particularly under drought. No significant differences were observed between low and high concentrations within each biostimulant. The 4% DBH treatment emerged as the most effective and economical option, increasing TChls by 23.9% (IR-1) and 58.7% (IR-2), TCars by 38.5% and 62.5%, SPAD by 17.7% and 41.5%, Fv/Fm by 15.5% and 25.5%, and PIABS by 30.9% and 60.4% compared to untreated controls.

**Table 5:** Response of photosynthetic parameters of *Phaseolus vulgaris* plants grown under two irrigation **regimes** [IR-1 (100% ETc) and IR-2 (60% ETc)] to foliar spraying with novel biostimulators (NBS); diluted lemon fruit juice (DLFJ) and diluted bee honey (DBH)

Treatments		Parameters				
Irrigation	NBS	TChls content	TCars content	SPAD	*Fv/Fm*	PI_ABS_
		mg g^−1^ FW				
IR-1	Control	1.42 ± 0.05^**c**^	0.26 ± 0.013^**c**^	36.1 ± 1.10^**c**^	0.58 ± 0.02^**c**^	16.2 ± 0.25^**c**^
	DLFJ-3%	1.56 ± 0.06^**b**^	0.30 ± 0.014^**b**^	39.3 ± 1.24^**b**^	0.62 ± 0.03^**b**^	18.8 ± 0.27^**b**^
	DLFJ-6%	1.54 ± 0.06^**b**^	0.31 ± 0.015^**b**^	39.4 ± 1.30^**b**^	0.62 ± 0.03^**b**^	18.6 ± 0.31^**b**^
	DBH-4%	1.76 ± 0.07^**a**^	0.36 ± 0.022^**a**^	42.5 ± 1.64^**a**^	0.67 ± 0.04^**a**^	21.2 ± 0.34^**a**^
	DBH-8%	1.72 ± 0.06^**a**^	0.35 ± 0.020^**a**^	42.8 ± 1.67^**a**^	0.68 ± 0.04^**a**^	21.1 ± 0.28^**a**^
IR-2	Control	0.92 ± 0.03^**g**^	0.16 ± 0.008^**g**^	25.3 ± 0.89^**g**^	0.47 ± 0.02^**f**^	10.1 ± 0.13^**f**^
	DLFJ-3%	1.04 ± 0.03^**f**^	0.19 ± 0.010^**f**^	28.4 ± 0.94^**f**^	0.51 ± 0.02^**e**^	11.8 ± 0.16^**e**^
	DLFJ-6%	1.15 ± 0.04^**e**^	0.21 ± 0.012^**e**^	31.1 ± 1.02^**e**^	0.55 ± 0.02^**d**^	14.0 ± 0.18^**d**^
	DBH-4%	1.46 ± 0.07^**c**^	0.26 ± 0.012^**c**^	35.8 ± 1.08^**c**^	0.59 ± 0.03^**c**^	16.2 ± 0.28^**c**^
	DBH-8%	1.44 ± 0.06^**c**^	0.27 ± 0.014^**c**^	36.4 ± 1.14^**c**^	0.59 ± 0.03^**c**^	16.0 ± 0.27^**c**^

Based on the two-way ANOVA conducted across all 10 treatment combinations and the LSD test, bars (mean ± standard error) labeled with similar letters in the same column did not differ significantly at a *p* ≤ 0.05 level of probability. Control (D-H_2_O); control plants sprayed with distilled water, TChls; total chlorophylls, TCars; total carotenoids, Fv/Fm; chlorophyll “a” fluorescence, PI_ABS_; photosynthetic performance index, and FW; fresh weight

### Effects of NBS on nutrient contents of drought-stressed *P. vulgaris* plants

**Fig. 1:**
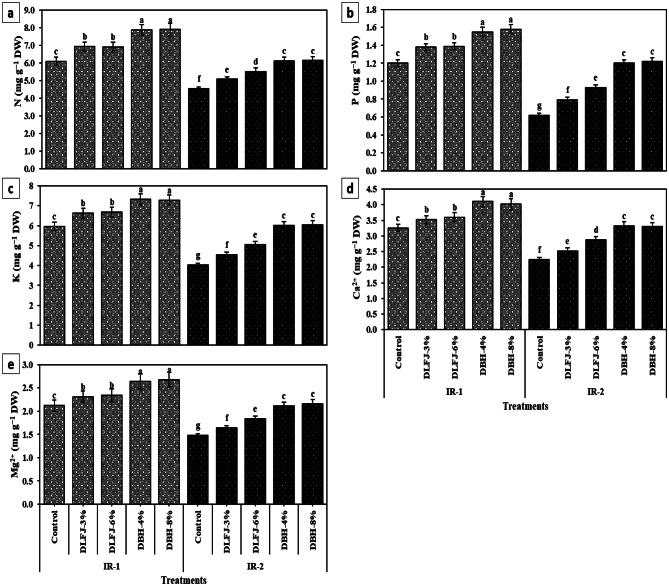
Response of macro-nutrient contents of *Phaseolus vulgaris* plants grown under two irrigation regimes [IRs; IR-1 (100% ETc) and IR-2 (60% ETc)] to foliar spraying with novel biostimulators (NBS); Diluted Lemon Fruit Juice (DLFJ) and Diluted Bee Honey (DBH). Based on the two-way ANOVA conducted across all 10 treatment combinations and the LSD test, bars (mean ± standard error) labeled with similar letters in the same column did not differ significantly at a *p* ≤ 0.05 level of probability. Cont. (D-H_2_O); control plants sprayed with distilled water, N; nitrogen, P; phosphorus, K^+^; potassium, Ca^2+^; calcium, Mg; magnesium, and DW; dry weight

**Fig. 2:**
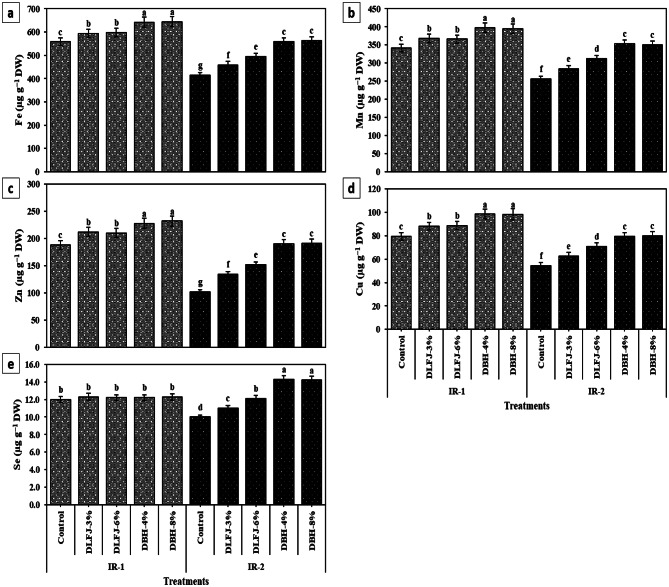
Response of micro-nutrient contents of *Phaseolus vulgaris* plants grown under two irrigation regimes [IRs; IR-1 (100% ETc) and IR-2 (60% ETc)] to foliar spraying with novel biostimulators (NBS); Diluted Lemon Fruit Juice (DLFJ) and Diluted Bee Honey (DBH). Based on the two-way ANOVA conducted across all 10 treatment combinations and the LSD test, bars (mean ± standard error) labeled with similar letters in the same column did not differ significantly at a *p* ≤ 0.05 level of probability. Cont. (D-H_2_O); control plants sprayed with distilled water, Fe; iron, Mn; manganese, Zn; zinc, Cu; copper, Se; selenium, and DW; dry weight

Drought stress (IR-2) significantly reduced the accumulation of key macro- and micronutrients in *P. vulgaris* leaves. Relative to IR-1, reductions under IR-2 ranged from 16.7% (Se) to 48.3% (P) (Figs. 1 and 2). Foliar application of both DLFJ and DBH improved nutrient levels under both irrigation regimes, with DBH treatments consistently outperforming DLFJ. Differences between low and high concentrations within each biostimulant were not statistically significant. DBH-4% proved most efficient, markedly enhancing nutrient contents under drought: P (+ 93.5%), Zn (+ 86.3%), K (+ 49.8%), Ca (+ 48.2%), and Fe (+ 35.3%), among others. This underscores its potential to mitigate nutrient depletion in water-limited conditions.

### Effects of NBS on leaf integrity, oxidative stress markers, and associated membrane damage of drought-stressed *P. vulgaris* plants

**Fig. 3:**
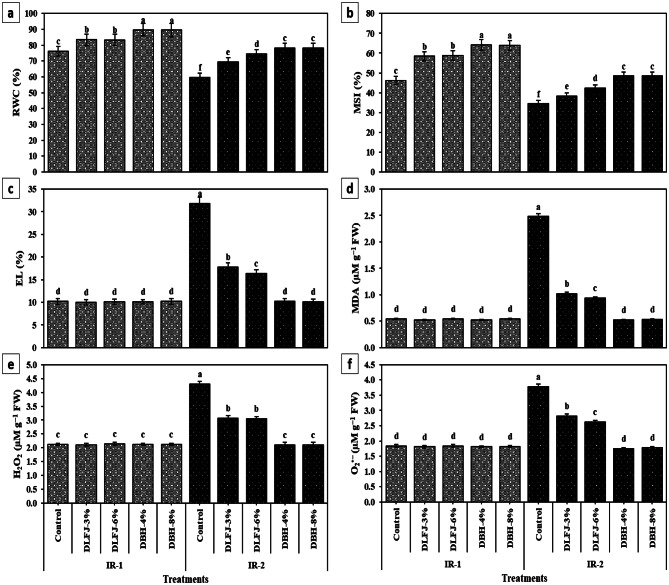
Response of leaf tissue integrity (relative water content; RWC and membrane stability index; MSI), oxidant levels, and oxidative damage of *Phaseolus vulgaris* plants grown under two irrigation regimes [IRs; IR-1 (100% ETc) and IR-2 (60% ETc)] to foliar spraying with novel biostimulators (NBS); Diluted Lemon Fruit Juice (DLFJ) and Diluted Bee Honey (DBH). Based on the two-way ANOVA conducted across all 10 treatment combinations and the LSD test, bars (mean ± standard error) labeled with similar letters in the same column did not differ significantly at a *p* ≤ 0.05 level of probability. Cont. (D-H_2_O); control plants sprayed with distilled water, MDA; malondialdehyde measured to evaluate membrane lipid peroxidation level, H_2_O_2_; hydrogen peroxide, O_2_^•−^; superoxide radical, and FW; fresh weight

Under drought conditions (IR-2), relative water content (RWC) and membrane stability index (MSI) decreased by 21.5% and 25.5%, respectively, while oxidative stress markers — electrolyte leakage (EL), malondialdehyde (MDA), hydrogen peroxide (H₂O₂), and superoxide anion (O₂^•−^) — increased by over 100% compared to full irrigation (IR-1) (Fig. 3). Foliar application of both biostimulants significantly improved RWC and MSI and suppressed oxidative damage. DBH treatments were more effective than DLFJ across irrigation regimes, with no significant differences between the 4% and 8% concentrations. DBH-4% yielded the most consistent results, particularly under drought: RWC and MSI increased by 30.8% and 40.7%, respectively, while EL, MDA, H₂O₂, and O₂^•−^ were reduced by 67.9%, 79.0%, 50.9%, and 116.0%, respectively, compared to untreated stressed plants.

### Effects of NBS on osmoprotectant (OSPs) and antioxidant levels of drought-stressed *P. vulgaris* plants

**Fig. 4:**
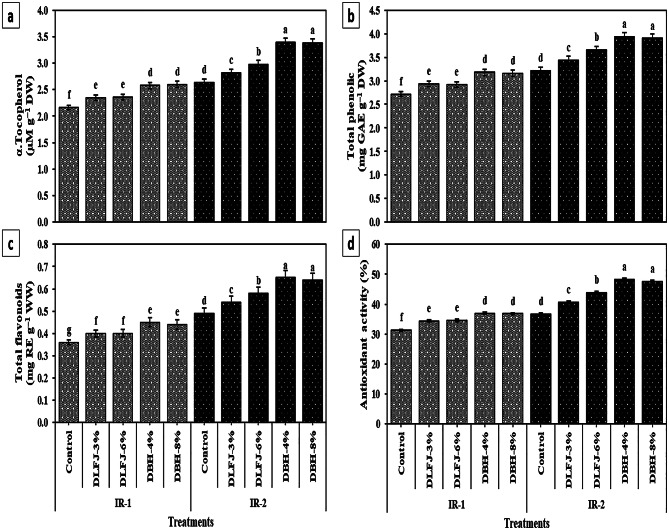
Response of phenolic compound contents and antioxidant activity of *Phaseolus vulgaris* plants grown under two irrigation regimes [IRs; IR-1 (100% ETc) and IR-2 (60% ETc)] to foliar spraying with novel biostimulators (NBS); Diluted Lemon Fruit Juice (DLFJ) and Diluted Bee Honey (DBH). Based on the two-way ANOVA conducted across all 10 treatment combinations and the LSD test, bars (mean ± standard error) labeled with similar letters in the same column did not differ significantly at a *p* ≤ 0.05 level of probability. Cont. (D-H_2_O); control plants sprayed with distilled water

Drought stress (IR-2) triggered increased accumulation of OSPs and antioxidants in *P. vulgaris* leaves, with significant elevations in proline (+ 37.5%), glycine betaine (GB, + 38.3%), and total antioxidant activity (TAA, + 17.3%) compared to well-watered plants (Table 6; Fig. 4). Foliar application of both DLFJ and DBH further enhanced these biochemical defenses under both irrigation regimes, with stronger effects observed under drought. DBH consistently outperformed DLFJ, and no significant differences were found between the two concentrations of either treatment. Among all treatments, DBH-4% was the most effective and economical, particularly under drought (IR-2), increasing TS sugars by 27.6%, GB by 23.0%, proline by 36.0%, AsA by 37.2%, GSH by 35.4%, α-tocopherol by 28.4%, total phenols by 22.4%, total flavonoids by 32.7%, and TAA by 31.3% compared to the untreated control.

**Table 6:** Response of osmoprotectant and non-enzymatic antioxidant levels of *Phaseolus vulgaris* plants grown under two irrigation regimes [IR-1 (100% ETc) and IR-2 (60% ETc)] to foliar spraying with novel biostimulators (NBS); diluted lemon fruit juice (DLFJ) and diluted bee honey (DBH)

Treatments		Parameters				
Irrigation	NBS	TS sugar content	GB content	Proline content	AsA level	GSH level
		mg g^−1^ DW	µM g^−1^ DW		µM g^−1^ FW	
IR-1	Control	16.4 ± 0.38^**d**^	21.4 ± 0.44^**e**^	106.8 ± 2.11^**e**^	1.44 ± 0.03^**g**^	0.47 ± 0.01^**e**^
	DLFJ-3%	16.2 ± 0.41^**d**^	21.5 ± 0.46^**e**^	108.2 ± 2.21^**e**^	1.58 ± 0.04^**f**^	0.55 ± 0.01^**d**^
	DLFJ-6%	16.4 ± 0.40^**d**^	21.7 ± 0.46^**e**^	106.8 ± 2.16^**e**^	1.56 ± 0.04^**f**^	0.54 ± 0.01^**d**^
	DBH-4%	17.0 ± 0.45^**d**^	26.6 ± 0.52^**d**^	128.2 ± 2.38^**d**^	1.74 ± 0.05^**e**^	0.63 ± 0.02^**c**^
	DBH-8%	18.8 ± 0.50^**c**^	26.4 ± 0.55^**d**^	130.6 ± 2.40^**d**^	1.74 ± 0.05^**e**^	0.64 ± 0.02^**c**^
IR-2	Control	19.2 ± 0.54^**c**^	29.6 ± 0.58^**c**^	146.8 ± 2.62^**c**^	1.88 ± 0.05^**d**^	0.65 ± 0.02^**c**^
	DLFJ-3%	21.4 ± 0.58^**b**^	31.9 ± 0.64^**b**^	164.2 ± 2.80^**b**^	2.06 ± 0.06^**c**^	0.71 ± 0.02^**b**^
	DLFJ-6%	21.6 ± 0.57^**b**^	31.8 ± 0.66^**b**^	165.8 ± 2.84^**b**^	2.22 ± 0.06^**b**^	0.72 ± 0.03^**b**^
	DBH-4%	24.5 ± 0.67^**a**^	36.4 ± 0.78^**a**^	199.6 ± 2.98^**a**^	2.58 ± 0.09^**a**^	0.88 ± 0.03^**a**^
	DBH-8%	24.8 ± 0.69^**a**^	36.4 ± 0.73^**a**^	198.8 ± 2.96^**a**^	2.54 ± 0.08^**a**^	0.86 ± 0.03^**a**^

Based on the two-way ANOVA conducted across all 10 treatment combinations and the LSD test, bars (mean ± standard error) labeled with similar letters in the same column did not differ significantly at a *p* ≤ 0.05 level of probability. Control (D-H_2_O); control plants sprayed with distilled water, TS-sugar; total soluble sugars, GB; glycine betaine, AsA; ascorbate, GSH; gluthathione, DW; dry weight, and FW; fresh weight

### Effects of NBS on the enzyme activities of drought-stressed *P. vulgaris* is plants

Under drought (IR-2), *P. vulgaris* plants exhibited elevated activities of antioxidant enzymes — SOD (+ 44.4%), CAT (+ 51.6%), APX (+ 44.6%), and GR (+ 45.7%) — alongside a 32.4% reduction in total protein content compared to full irrigation (Table 7). While biostimulant application had minimal effect under well-watered conditions (IR-1), all treatments significantly enhanced protein levels and enzyme activities under drought. DBH treatments showed stronger effects than DLFJ, with no statistical difference between the low and high concentrations. The 4% DBH treatment was the most effective, increasing protein content by 50.8%, SOD by 18.6%, CAT by 41.5%, APX by 21.1%, and GR by 19.5% compared to untreated drought-stressed plants.

**Table 7:** Response of enzyme activities of *Phaseolus vulgaris* plants grown under two irrigation regimes [IR-1 (100% ETc) and IR-2 (60% ETc)] to foliar spraying with novel biostimulators (NBS); diluted lemon fruit juice (DLFJ) and diluted bee honey (DBH)

Treatments		Parameters				
Irrigation	NBS	SOD activity	CAT activity	APX activity	GR activity	Sol-protein content
		Unit g^−1^ protein				mg g^−1^ DW
IR-1	Control	32.4 ± 0.68^**d**^	12.4 ± 0.22^**d**^	18.4 ± 0.17^**d**^	16.2 ± 0.24^**d**^	122.2 ± 2.32^**a**^
	DLFJ-3%	32.8 ± 0.70^**d**^	12.6 ± 0.22^**d**^	18.4 ± 0.20^**d**^	16.8 ± 0.26^**d**^	126.0 ± 2.34^**a**^
	DLFJ-6%	32.4 ± 0.67^**d**^	12.4 ± 0.25^**d**^	18.9 ± 0.23^**d**^	16.9 ± 0.26^**d**^	126.2 ± 2.48^**a**^
	DBH-4%	33.2 ± 0.68^**d**^	12.5 ± 0.24^**d**^	18.6 ± 0.20^**d**^	16.8 ± 0.24^**d**^	126.3 ± 2.55^**a**^
	DBH-8%	32.8 ± 0.70^**d**^	12.4 ± 0.22^**d**^	18.7 ± 0.22^**d**^	17.0 ± 0.25^**d**^	127.0 ± 2.57^**a**^
IR-2	Control	46.8 ± 0.92^**c**^	18.8 ± 0.28^**c**^	26.6 ± 0.30^**c**^	23.6 ± 0.29^**c**^	82.6 ± 1.80^**c**^
	DLFJ-3%	49.9 ± 0.96^**b**^	20.6 ± 0.33^**b**^	29.6 ± 0.40^**b**^	25.4 ± 0.32^**b**^	94.4 ± 1.92^**b**^
	DLFJ-6%	50.2 ± 0.98^**b**^	21.2 ± 0.37^**b**^	30.0 ± 0.45^**b**^	25.6 ± 0.33^**b**^	95.0 ± 1.96^**b**^
	DBH-4%	55.5 ± 1.12^**a**^	26.6 ± 0.45^**a**^	32.2 ± 0.51^**a**^	28.2 ± 0.39^**a**^	124.6 ± 2.28^**a**^
	DBH-8%	55.4 ± 1.08^**a**^	26.4 ± 0.43^**a**^	32.4 ± 0.52^**a**^	28.2 ± 0.40^**a**^	123.8 ± 2.26^**a**^

Based on the two-way ANOVA conducted across all 10 treatment combinations and the LSD test, bars (mean ± standard error) labeled with similar letters in the same column did not differ significantly at a *p* ≤ 0.05 level of probability. Control (D-H_2_O); control plants sprayed with distilled water, SOD; superoxide dismutase, CAT; catalase, APX; ascorbate peroxidase, GR; glutathione reductase, Sol-protein; soluble protein, and DW; dry weight

### Effects of NBS on growth and yield traits and quality of drought-stressed *P. vulgaris* plants

**Fig. 5:**
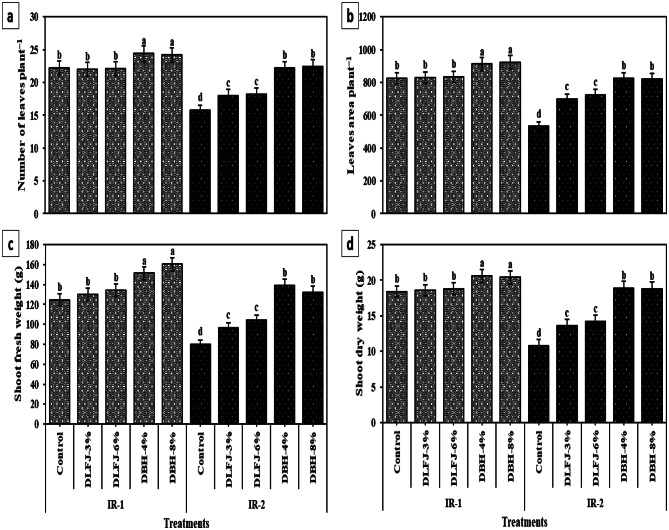
Response of growth traits of *Phaseolus vulgaris* plants grown under two irrigation regimes [IRs; IR-1 (100% ETc) and IR-2 (60% ETc)] to foliar spraying with novel biostimulators (NBS); Diluted Lemon Fruit Juice (DLFJ) and Diluted Bee Honey (DBH). Based on the two-way ANOVA conducted across all 10 treatment combinations and the LSD test, bars (mean ± standard error) labeled with similar letters in the same column did not differ significantly at a *p* ≤ 0.05 level of probability. Cont. (D-H_2_O); control plants sprayed with distilled water

Drought stress (IR-2) significantly reduced *P. vulgaris* growth and yield performance, with leaf number, leaf area, shoot biomass, pod count, and total yield declining by 28–41% relative to well-watered controls (Fig. 5; Table 8). In contrast, fiber (+ 37.3%), vitamin C (+ 41.9%), and nitrate (+ 96.0%) contents increased under stress, reflecting altered pod quality. Biostimulant application notably improved all growth, yield, and quality metrics under both irrigation regimes, with stronger effects under drought. DBH treatments outperformed DLFJ, and no significant differences were detected between low and high concentrations within each type. DBH-4% was the most effective treatment, increasing leaf area by 54.9%, shoot fresh weight by 73.6%, green pod yield by 54.0%, and protein content by 41.8% under drought. Concurrently, it reduced fiber (− 27.5%) and nitrate content (− 53.5%), enhancing pod nutritional quality.

**Table 8:** Response of green pod yield components and green pod quality traits of *Phaseolus vulgaris* plants grown under two irrigation regimes [IRs; IR-1 (100% ETc) and IR-2 (60% ETc)] to foliar spraying with novel biostimulators (NBS); diluted lemon fruit juice (DLFJ) and diluted bee honey (DBH)

Treatments		Parameters							
Irrigation	NBS	Number of green pods plant^−1^	Pod weight plant^−1^	Green pods yield ha^−1^	Protein content		Fibers content	Vitamin C content	NO_3_^−^ content
			g	ton	%			mg g^−1^ FW	mg g^−1^ DW
IR-1	Control	21.4 ± 1.06^**b**^	138 ± 6.8^**b**^	3.31 ± 0.16^**b**^	21.4 ± 1.12^**c**^	4.24 ± 0.34^**c**^		0.86 ± 0.04^**d**^	2.48 ± 0.14^**c**^
	DLFJ-3%	22.0 ± 1.10^**b**^	140 ± 7.0^**b**^	3.36 ± 0.18^**b**^	23.0 ± 1.18^**b**^	4.20 ± 0.35^**c**^		0.88 ± 0.04^**d**^	2.21 ± 0.12^**d**^
	DLFJ-6%	22.2 ± 1.12^**b**^	138 ± 7.0^**b**^	3.34 ± 0.18^**b**^	23.2 ± 1.19^**b**^	4.22 ± 0.34^**c**^		0.88 ± 0.05^**d**^	2.19 ± 0.11^**d**^
	DBH-4%	25.2 ± 1.22^**a**^	172 ± 7.8^**a**^	4.13 ± 0.22^**a**^	24.8 ± 1.24^**a**^	4.22 ± 0.33^**c**^		0.90 ± 0.05^**d**^	1.98 ± 0.10^**e**^
	DBH-8%	24.8 ± 1.18^**a**^	176 ± 7.9^**a**^	4.22 ± 0.24^**a**^	24.9 ± 1.32^**a**^	4.20 ± 0.34^**c**^		0.89 ± 0.05^**d**^	1.94 ± 0.09^**e**^
IR-2	Control	14.6 ± 0.76^**d**^	91 ± 4.8^**d**^	2.15 ± 0.12^**d**^	15.8 ± 0.80^**e**^	5.82 ± 0.42^**a**^		1.22 ± 0.06^**c**^	4.86 ± 0.28^**a**^
	DLFJ-3%	17.9 ± 0.97^**c**^	114 ± 5.7^**c**^	2.72 ± 0.14^**c**^	19.8 ± 0.96^**d**^	4.71 ± 0.39^**b**^		1.38 ± 0.06^**b**^	3.22 ± 0.22^**b**^
	DLFJ-6%	18.4 ± 0.98^**c**^	118 ± 6.1^**c**^	2.80 ± 0.14^**c**^	20.2 ± 1.06^**d**^	4.66 ± 0.37^**b**^		1.41 ± 0.07^**b**^	3.26 ± 0.20^**b**^
	DBH-4%	21.9 ± 1.18^**b**^	140 ± 6.9^**b**^	3.31 ± 0.16^**b**^	22.4 ± 1.18^**c**^	4.22 ± 0.35^**c**^		1.72 ± 0.07^**a**^	2.26 ± 0.12^**d**^
	DBH-8%	21.6 ± 1.14^**b**^	142 ± 7.0^**b**^	3.33 ± 0.17^**b**^	22.0 ± 1.14^**c**^	4.20 ± 0.35^**c**^		1.74 ± 0.08^**a**^	2.28 ± 0.12^**d**^

Based on the two-way ANOVA conducted across all 10 treatment combinations and the LSD test, bars (mean ± standard error) labeled with similar letters in the same column did not differ significantly at a *p* ≤ 0.05 level of probability. Control; control plants sprayed with distilled water and NO_3_^−^; nitrate

## Discussion

As with most crop plants, drought stress (DrS) affects vegetable crop plants, especially in arid climates, including Egypt (Alghamdi et al. 2022; Morsi et al. 2023). It significantly restricts plant performance, impacting growth and yield, and disrupts various metabolic processes by inducing excessive ROS production, severely affecting plant development and yield quality. To counteract DrS, plants activate numerous particular mechanisms, including ion homeostasis, osmotic adjustment, and stimulation of antioxidant protection (Seleiman et al. 2021; Yang et al. 2021). However, these self-defences are often insufficient under prolonged DrS, necessitating support through exogenous applications to maintain sustainable agricultural productivity (Abd El-Mageed et al. 2023; Hemida et al. 2024). Diluted lemon fruit juice (DLFJ) and bee honey (DBH) have been identified as highly efficient novel biostimulants (NBS), which contain several essential bioactive components (EBCs) (Semida et al. 2019; Rady et al. 2021; Alghamdi et al. 2022; Belal et al. 2023). These NBS can enhance drought stress resistance by helping hormonal and nutritional balance, regulating antioxidants, osmotic defenders, and gene expression regulation, thereby improving plant productivity, growth, and quality in *Phaseolus vulgaris* plants (Alghamdi et al. 2022; Belal et al. 2023). Therefore, the potential role of foliar sprays of DLFJ (DLFJ-3% and DLFJ-6%) and DBH (DBH-4% and DBH-8%) under two watering regimes [100% ETc (IR-1) and 60% ETc (IR-2)] in enhancing photosynthesis, nutrient uptake/content, leaf tissue integrity, lipid peroxidation markers, osmoprotectants (OSPs), and different antioxidant activities, growth, yield, and quality traits of *P. vulgaris* plants was examined.

Analysis of lemon fruit juice and raw clover honey indicates that they are rich in EBCs (Table 3), making both lemon juice and bee honey valuable biostimulants. Therefore, the use of their dilutions at the specified concentrations (DLFJ-3% and DBH-4%), is an effective approach for rebalancing inorganic nutrients. This approach also optimizes the efficacy of various antioxidants and OSPs as protective measures for *P. vulgaris* plants facing DrS challenges.

DrS considerably decreased TChls and TCars contents, SPAD, *Fv/Fm*, and PI_ABS_ of *P. vulgaris* plants. This finding supports previous results (Dawood and Abeed 2020; Abeed et al. 2021; Alghamdi et al. 2022). Numerous factors might account for this negative finding: pigment collapse from gathered ROS (Mathobo et al. 2017), diminished pigment synthesis (Seleiman et al. 2021) due to augmented action of pigment-degrading chlorophyllase activity (Trifunović-Momčilov et al. 2021), and/or distractions in the production of proteins vital to chlorophyll (Oguz et al. 2022). Although DrS reduces photosynthetic pigments and efficiency (Alghamdi et al. 2022; Pandey et al. 2023; Nasiri et al. 2024), the use of NBS; DLFJ, and DBH, in this research markedly improved photosynthetic indices of *P. vulgaris* plants. Under IR-2, foliar treatment with DBH-4% (best treatment) significantly enhanced the photosynthetic parameters of *P. vulgaris* plants, outperforming DLFJ-3% (and DLFJ-6%). It is possible that the NBS, rich EBCs, and pigments like carotenoids could be captivated by the vegetation, thus increasing their pigment arrangement and overall resistance (Rakkammal et al. 2023). The NBS may also initiate gene accountable for pigment production in vegetation (Abou-Sreea et al. 2021; Franzoni et al. 2022).

Under IR-2, nutrient (N, P, K^+^, Ca^2+^, Mg, Fe, Mn, Zn, Cu, and Se) contents were lower in *P. vulgaris* plants than those gathered under IR-1. Thus, nutrient insufficiency in vegetation caused by DrS is attributed to osmotic effects and/or soil DrS, which disrupts nutrient availability, transfer, and uptake (Kapoor et al. 2020), causing nutrient deficiency in *P. vulgaris* plants. Nevertheless, foliar treatment with DBH-4% (or DBH-8%) had higher nutrient (N, P, K^+^, Ca^2+^, Mg, Fe, Mn, Zn, Cu, and Se) content than those in plants sprayed with DLFJ-3% (or DLFJ-6%). This finding may be due to NBS enhancing the surface area for root uptake, resulting from an increased root system capacity and/or the addition of biostimulants that help balance osmotic stress in plant cells. As a result, this improves the nutrient status and maintains water uptake and cell turgor (Halpern et al. 2015; Singh et al. 2015).

DrS considerably decreased RWC and MSI in *P. vulgaris* leaves. Numerous scientists have stated a vivid drop-in leaf RWC and MSI under DrS (Dwivedi et al. 2018; Sakya et al. 2018). RWC is an appropriate dimension of plant water position as a functional significance of cellular DrS (Abd El-Mageed et al. 2017). MSI is a broadly used standard to measure crop DrS tolerance, meanwhile, DrS causes water loss from plant flesh, damaging both membrane function and structure (Jha and Subramanian 2014). However, foliar application of DLFJ or DBH significantly increased leaf RWC and MSI, which were higher under IR-1 than under IR-2. Overall, the application of NBS under IR-2, in this study was effective in improving leaf integrity greater than that under IR-1 (normal circumstances), agreeing with (Abd El-Mageed et al. 2017) and (Peña Calzada et al. 2022).

In this study, DrS considerably augmented oxidant stress indicators (H_2_O_2_ and O_2_^•–^ levels) and oxidative damage (EL and MDA levels) in *P. vulgaris* plants. Like the present study, Rady et al. (Rady et al. 2021) found an upsurge in these oxidative damage indicators in *Vicia faba* plants under DrS. Exposure to DrS results in the overproduction of various types of reactive oxygen species (ROS), including singlet oxygen (^1^O_2_), superoxide radicals (O_2_^•−^), hydrogen peroxide (H_2_O_2_) and hydroxyl radical (^•^OH‾). These substances are produced in amounts that exceed the body’s ability to metabolize them, leading to potential reactions with multiple cellular components and causing severe oxidative damage (Bhuiyan et al. 2019; Sohag et al. 2020). In this research, foliar treatment with DLFJ and DBH significantly suppressed oxidative stress and damage in *P. vulgaris* plants. Rady et al. (Rady et al. 2021) and Tarfayah et al. (Tarfayah et al. 2023) also observed that, under DrS, biostimulant applications (DBH, or silymarin-enriched DBH) considerably reduced EL, MDA, H_2_O_2_, and O_2_^•−^ levels in favor of plant photosynthetic efficiency and growth.

In addition, DrS affected the levels of OSPs (total soluble sugars, GB, and proline) and antioxidants (AsA, GSH, and proline) in *P. vulgaris* plants. However, foliar application with NBS like DLFJ and DBH significantly improved the levels of OSPs and antioxidants under IR-2. The GSH and AsA play vital roles in ROS detoxification by the “AsA-GSH cycle”, helping protect cell biomolecules and organelles from oxidative stress (Hasanuzzaman et al. 2021). AsA is a durable electron donor and antioxidant, so it deactivates ROS by electron delocalization. It similarly contributes to the biosynthetic passageway of certain plant hormones (Akram et al. 2017). In disparity, GSH vanishes ROS through the “AsA-GSH cycle” and withstands redox stress (Hasanuzzaman et al. 2020). The constructive condition offered by DBH (as the best treatment) protected plants from the harmful effects of DrS by altering osmotic pressure and scavenging ROS (Semida et al. 2019; Khalid and Aftab 2020). Moreover, NBS treatment controls diverse genes, which could modify the osmotic capability to sustain cell amplification by gathering osmotically energetic solutes such as TS sugars, GB, and proline contents (Rady et al. 2021).

Common beans contain remarkably high phytochemical contents, including EBCs like α-tocopherol, phenolics, flavonoids, and antioxidant composites (Kumar et al. 2024; Myrtsi et al. 2024). These EBCs display therapeutic and health benefits together with vitamin deficiencies, cholesterol-lowering, antioxidant, anti-cancer, anti-inflammatory, and anti-diabetic potential (Bai et al. 2023). In this research, DrS decreases α-tocopherol, total phenol and flavonoid levels, and antioxidant activity in *P. vulgaris* plants. This finding is in line with (Kusvuran and Dasgan 2017), who also witnessed the decrease in these EBCs in *P. vulgaris* plants under DrS. However, under IR-2, foliar treatment with NBS, especially DBH-4%, optimized the levels of these EBCs and antioxidant activity, which agrees with (Alghamdi et al. 2022). DLFJ and DBH are natural solutions containing different EBCs (Table 3) (Semida et al. 2019; Abou-Sreea et al. 2021; Alghamdi et al. 2022; Rady et al. 2023a, b). These papers signalize that DLFJ and DBH function as active antioxidants that scavenge ROS by preventing the auto-oxidation of flavonoids, which serves as a defense against DrS-induced oxidative stress (Isla et al. 2011; Makni et al. 2018). In addition, the EBCs found in DLFJ and DBH can raise stress tolerance in crop plants.

In this work, under DrS, enzyme activities (e.g., SOD, CAT, APX, and GR) were enhanced in *P. vulgaris* plants, while protein levels decreased. These findings are consistent with the results of (Alghamdi et al. 2022). As protection mechanisms of tolerant genotypes contrary to stress, these enzymes are commonly energetic in decreasing H_2_O_2_ to water in mitochondria and chloroplasts, thus purifying them (Mishra et al. 2023). APX protects plants during stress by consuming AsA as an electron donor to eliminate deadly H_2_O_2_ (Li 2023). GR activity is raised under severe DrS to catalyze the NADP-dependent decrease of GSSG to produce GSH to eliminate dioxygen under stress situations (Hasanuzzaman et al. 2017). The redevelopment of GSH from GSSG by GR is significant in eliminating ROS (Dorion et al. 2021). SOD is measured as unique enzymatic arrangements to scavenge stress-produced O_2_^•−^ in plants (Rajput et al. 2021). An additional enzyme, CAT works synergistically with SOD to hinder the expansion of the destructive ROS; O_2_^•−^ and H_2_O_2_ (Wang et al. 2018). Further activation of the enzyme activities examined in plants by NBS could suppress the extent of stress-stimulated damage (Mishra et al. 2023). Consequently, the enzyme activities examined could play substantial roles in rapid defense reactions in plant cells against oxidative stress (Llauradó Maury et al. 2020). Under IR-2, foliar treatment with DLFJ and DBH optimized the protein content and enzyme activities examined in *P. vulgaris* plants, similar to those of (Alghamdi et al. 2022).

*Phaseolus vulgaris* growth, yield, and green pod quality traits (e.g., protein, fiber, vitamin C, and NO_3_^−^ contents) were considerably lower under IR-2 than under IR-1. However, foliar application of NBS (DLFJ and DBH) significantly increased these parameters. Under DrS, optimization of growth, productivity, and pod quality of *P. vulgaris* plants was achieved by NBS, particularly DBH-4%. This is due to the NBS stimulating multiple mechanisms (B-group vitamins, antioxidants, OSPs, and nutrients). These mechanisms help plants restore their development, growth, and yield quality (pod protein, vitamin C, NO_3_^−^, and fiber contents) due to DrS mitigation. The trend of this finding is similar to that of (Saxena et al. 2010; Semida et al. 2019; Desoky et al. 2020; Klimek-Szczykutowicz et al. 2020; Rady et al. 2021).

Previous research demonstrates the efficacy of biostimulants in enhancing drought tolerance in legumes. For instance, (Rady and Mohamed 2015) reported that foliar application of *Moringa oleifera* leaf extract improved photosynthetic efficiency and OSPs accumulation in P. vulgaris under DrS, similar to the enhancements observed in our study with DBH and DLFJ. However, while moringa-based biostimulants primarily exert their effects by regulating phytohormones and inducing activation of antioxidant enzymes, DBH and DLFJ provide a broader spectrum of OSPs (e.g., soluble sugars, glycine betaine, and proline), which may contribute to more robust drought resilience mechanisms. Additionally, our results are consistent with (Pascual et al. 2018), who found that seaweed-derived biostimulants significantly improved nutrient uptake and stress tolerance in legumes. However, our study uniquely demonstrates that DBH-4% significantly reduced oxidative stress markers [malondialdehyde (MDA) and hydrogen peroxide (H₂O₂)] while increasing antioxidant enzyme activities (SOD, CAT, APX, and GR) under DrS. This suggests that the polyphenolic and enzymatic composition of DBH may offer a more direct ROS-scavenging effect compared to plant-based extracts. Compared to synthetic biostimulants such as salicylic acid (SA) or commercial humic substances, which have been widely used to enhance drought tolerance in legumes (Desoky et al. 2021), our approach provides an eco-friendly and cost-effective alternative. While synthetic treatments often target specific signaling pathways, the multifunctional bioactive composition of DBH-4% enhances multiple physio-biochemical defense mechanisms simultaneously. Furthermore, the higher increase in green pod yield observed with DBH-4% under DrS surpasses previous reports using synthetic treatments, highlighting the practical applicability of food-based biostimulants in sustainable agriculture.

The results from the DBH treatments were superior to those from the DLFJ treatments. Furthermore, there was no significant difference between the results of the DBH-4% and DBH-8% treatments. Therefore, the DBH-4% treatment is recommended as a cost-effective option for sustaining the production and quality of *P. vulgaris* under dry conditions (60% ETc).

The results of this study indicate that the recommended DBH-4% treatment is the most effective in improving DrS tolerance. However, the challenges in biostimulant science have grown because the composition and concentration of active substances in the original plant materials can be affected by various factors. These factors include the location, growing conditions, season, plant species, variety, specific plant organ, and the growth phase. Similarly, the response of the target crop is expected to vary across different crops and environments. One solution to this problem is to source the raw materials for the biostimulant under highly regulated conditions. Leading seaweed producers and manufacturers of fermentation-based products have successfully implemented harvesting and production processes that ensure consistent product performance over time. Ensuring that a product has a consistent response does not guarantee that it is optimized for biological effectiveness. To tackle these challenges, advancements in omics approaches will be essential in accelerating the discovery of how bioactive compounds work and in optimizing their applications (Yakhin et al. 2017). Future research should focus on the synergistic effects between microbial and non-microbial biostimulants, as well as the design and formulation of functional biostimulants. This approach aims to create efficient biostimulant products with specific properties that enhance yield and resource use efficiency (Rouphael et al. 2025).

## Conclusions

This study demonstrates the efficacy of diluted lemon fruit juice (DLFJ) and bee honey (DBH) as novel biostimulants in mitigating drought stress in *Phaseolus vulgaris*. Both biostimulants enhanced key physiological parameters, including photosynthetic efficiency, relative water content, and antioxidant defense systems. Among the treatments, DBH at a 4% concentration was the most effective, significantly improving growth, nutrient uptake, and yield under both full and limited irrigation. The bioactive compounds in bee honey likely contributed to these positive outcomes by reducing oxidative stress and enhancing nutrient absorption. These findings align with existing research on the role of biostimulants in enhancing plant tolerance to abiotic stressors and offer a sustainable, eco-friendly alternative to chemical agro-inputs. While this study highlights the potential of DBH and DLFJ, further research is needed to explore their effects on soil microbial communities and nutrient cycling with repeated field use. In addition, broader applications and economic feasibility of DBH and DLFJ should be applied on a larger scale. Using natural biostimulants like DBH presents a promising strategy for improving crop resilience and productivity in drought-prone regions.

## Data Availability

The datasets used and/or analysed during the current study are available from the corresponding author upon reasonable request.

## Abbreviations

Cd: Cadmium
DPPH: 2,2-diphenyl-1-picrylhydrazyl
DrS: Drought stress
ROS: Reactive oxygen species
LFJ: Lemon fruit juice
DLFJ: Diluted lemon fruit juice
BH: Bee honey
DBH: Diluted bee honey
NBS: Novel biostimulants
EBCs: Essential bioactive components
ECe: Soil extract electrical conductivity
NO_3_^−^: Nitrate
EC: Electrical conductivity
NaClO: Sodium hypochlorite
D-H_2_O: Deionized water
TSS: Total soluble solids
CaH_6_O_9_P_2_: Calcium superphosphate
K_2_SO_4_: Potassium sulfate
L^−1^: Per liter
ha^−1^: Per hectare
DLFJ-3%: 3% diluted lemon fruit juice
DLFJ-6%: 6% diluted lemon fruit juice
DBH-4%: 4% diluted bee honey
DBH-8%: 8% diluted bee honey
IR: Irrigation regime
ARC: Agricultural Research Center
ETc: Crop evapotranspiration
PAR: Photosynthetically active radiation
Chl: Chlorophyll
TChls: Total chlorophyll content
TCars: Total carotenoids content
SPAD: Soil plant analysis development
Fv/F0: Photochemical quantum yield
Fv/Fm: Chl “a” fluorescence; PSII photochemical efficiency
PI_ABS_: PSII performance index
IR-1: Irrigation at 100% ETc
IR-2: Irrigation at 60% ETc
RWC: Relative water content
MSI: Membrane stability index
EL: Electrolyte leakage
O_2_^•−^: Superoxide anion
H_2_O_2_: Hydrogen peroxide
MDA: Malondialdehyde
GA_3_: Gibberellic acid
OSPs: Osmoprotectants
GB: Glycine betaine
AsA: Ascorbate
GSH: Reduced glutathione
TS sugars: Total soluble sugars
NADP: Nicotinamide adenine dinucleotide phosphate
SOD: Superoxide dismutase
APX: Ascorbate peroxidase
GR: Glutathione reductase
GSSG: Oxidized glutathione
CAT: Catalase
N: Nitrogen
P: Phosphorus
K: Potassium
Ca: Calcium
Mg: Magnesium
Fe: Iron
Mn: Manganese
Zn: Zinc
Cu: Copper
Se: Selenium
NaHCO_3_: Sodium bicarbonate
IC_50_: Half maximal inhibitory concentration
HNO_3_: Nitric acid
HCl: Hydrochloric acid
µg g^−1^: Microgram per gram
µM g^−1^: Micromole per gram
FW: Fresh weight
HPLC: High-performance liquid chromatography
DW: Dry weight
OD: Optical density
NaOH: Sodium hydroxide
NBT: Nitro-blue tetrazolium
TAA: Total antioxidant activity
